# The Clinicopathological Characteristics of Young-Onset Versus Adult-Onset Colorectal Cancer: A Tertiary Hospital-Based Study

**DOI:** 10.21315/mjms2024.31.1.17

**Published:** 2024-02-28

**Authors:** Rilwanu Isah Tsamiya, Siti Norasikin Mohd Nafi, Nur Asyilla Che Jalil, Anani Aila Mat Zin

**Affiliations:** 1 Department of Pathology, School of Medical Sciences, Universiti Sains Malaysia, Kelantan, Malaysia; 2 Hospital Universiti Sains Malaysia, Kelantan, Malaysia

**Keywords:** early-onset, late-onset, colorectal cancer, clinicopathological, Malaysia

## Abstract

**Background:**

The prevalence of colorectal cancer (CRC) among young individuals is rising worldwide, especially in Malaysia. Investigations are currently employed to distinguish the features of young-onset CRC (YOCRC) from adult-onset CRC (AOCRC). This study aimed to compare the characteristics of patients with YOCRC and AOCRC diagnosed at Hospital Universiti Sains Malaysia (HUSM).

**Methods:**

This was a retrospective study of CRC cases from January 2013 to December 2021. The details of YOCRC (< 50 years old) and AOCRC (≥ 50 years old) patients were retrieved from the laboratory system and medical records. The Pearson’s chi-square test, Fisher’s exact test and multiple logistic regression were used to compare the AOCRC and YOCRC cases. Statistical significance was defined at a *P-*value of ≤ 0.05.

**Results:**

The AOCRC (254/319, 79.6%) was more prevalent than YOCRC (65/319, 20.4%), with a predominance of males (53.9%) and Malay sub-population (90.2%). AOCRC and YOCRC shared similarities in left-sided location, high occurrence of adenocarcinoma with moderately differentiated histology and advanced stage of diagnosis. More patients with YOCRC (23.1%) had a family history of cancer than patients with AOCRC. YOCRC also differed from AOCRC by having more specific histological subtypes, such as mucinous adenocarcinoma (15.4%) and signet ring carcinoma (6.2%). In addition, patients with YOCRC commonly presented with a low density of tumour-infiltrating lymphocytes (TILs) (60%). Multiple logistic regression showed a family history of CRC (adjusted odds ratio [AOR] = 3.75, *P* = 0.003) and histological type (AOR = 15.21, *P* < 0.001) are more likely to cause YOCRC than diabetes (AOR = 0.06, *P* < 0.001) and hypertension (AOR = 0.14, *P* < 0.001) comorbidities, which are associated with AOCRC.

**Conclusion:**

Our descriptive study presented the epidemiological and histopathological characteristics of AOCRC and YOCRC in HUSM, providing current information on distinguishing features between the groups.

## Introduction

Despite breakthroughs in understanding colorectal cancer (CRC) pathogenesis and the continuous evolvement of new treatment modalities, CRC remains one of the significant public health burdens (1, 2). CRC is the third most diagnosed malignancy in recent years and the second leading cause of cancer death globally (3–5). The GLOBOCAN report in 2020 has estimated that there would be 1,931,590 million new CRC cases worldwide (10% of all cancer diagnoses) and 935,173 million CRC-related deaths at 9.4% of all cancer-related fatalities (6). Therefore, if no crucial measures are taken to reverse the trend, the global number of new CRC cases will reach 3.2 million by 2040 (7). As a developing country in Southeast Asia, Malaysia has seen an increasing trend of CRC for years. Malaysian National Cancer Registry has reported an increased incidence of CRC from 13.2% in 2007–2011 to 13.5% in 2012–2016 (8). Additionally, according to International Agency for Research on Cancer, Malaysia was expected to have 6,597 (13.6%) new cases and a death rate of 3,420 (11.6%) for all sexes and ages in 2020 (9).

Previous studies had compared annual incidence, family history of CRC, clinicopathological features, and the overall survival rate in young-onset CRC (YOCRC) < 50 years old and adult-onset CRC (AOCRC) ≥ 50 years old (10–12). AOCRC, also called late-onset CRC, was reported to have prominence on the proximal colon with a declining rate due to the implemented screening programmes among these adult patients (13, 14). Meanwhile, young-onset CRC (YOCRC), also called early-onset CRC, had a global rise in incidence and often presented with more advanced disease at diagnosis (15–17). Furthermore, YOCRC demonstrated particular molecular and clinical markers associated with a different biologic phenotype from AOCRC (18, 19). For example, tumour-infiltrating lymphocytes (TILs) are linked to the tumour’s immunological status. Several studies have found the density of TILs to be a positive indicator in the prognosis of several cancers, including CRC (20, 21). TILs differ between patients with YOCRC and AOCRC, with higher TILs density linked with improved disease-specific and overall survival outcomes in patients with CRC (22–24).

A recent case report has documented two cases of YOCRC in HUSM, both of which have no family history or predisposing risk factors, suggesting that it is challenging to obtain early detection of CRC in this population of patients (25). Due to this, the current investigation aims to establish the clinicopathological features of YOCRC that would differentiate them from the late-onset subgroup. These data will favour early disease diagnosis in the future by increasing early suspicion of CRC in young people. The patient’s prognosis will be significantly improved by early diagnosis and intervention. To the best of our knowledge, there is a lack of current literature highlighting the comparison of YOCRC’s and AOCRC’s epidemiological and histopathological characteristics in our tertiary hospital. This study aimed to compare the characteristics of patients with YOCRC and AOCRC diagnosed in HUSM.

## Methods

This study was a retrospective assessment of CRC cases in the Department of Pathology in HUSM from January 2013 to December 2021. This tertiary hospital is located in Kelantan, one of the states in the Northeastern Peninsular of Malaysia (26). The Laboratory Information System and patients’ medical records were used to obtain patients’ information. Patients with CRC with complete data were included in the study, while those with incomplete information were excluded. In this study, we classified YOCRC as CRC below 50 years old, while AOCRC as CRC of 50 years old and above, as described in previous studies (27–29).

Data on age, gender, ethnicity, family history of CRC, type of specimen, tumour site, initial symptom, comorbidities and carcinoembryonic antigen (CEA) levels were recorded. Tumours in the transverse colon, hepatic flexure, ascending colon or cecum were classified as proximal colon tumours. In contrast, the tumours at the splenic flexure, descending colon and sigmoid colon were classified as distal colon tumours.

The histological type of CRC and its histological grade were obtained from the pathological reports. Data on TNM staging were also retrieved from similar reports, which included the primary tumour (T), regional lymph nodes (N) and distant metastasis (M) staging classification (30). They were further classified into local stages (stages I and II) and advanced stages (stages III and IV) (31). TILs density scores (high versus low) were also obtained from the reports and these values were previously validated on haematoxylin and eosin (H&E) stained sections by utilising the recommendation from the International TILs Working Group (32).

GraphPad Prism version 9.4.1 was used to analyse all the data (GraphPad Software Inc., United States). The mean and standard deviation were used to calculate continuous variables, whereas the proportions and percentages were used to summarise categorical variables. The Pearson’s chi-squared and Fisher’s exact tests were performed to assess the differences in demographic and clinicopathological parameters of young and adult CRCs. Significant factors associated with comparing YOCRC and AOCRC were identified using multiple logistic regression analysis. The predictors’ crude odds ratios were determined using simple logistic regression in the logistic regression (LR) analysis. Those with *P*-values below 0.25 were deemed significant factors and included in the multiple LR analysis to determine the predictors’ adjusted odds ratios (AOR). The forward LR and back LR methods were used in the multiple logistic regression and the final model was run using the Enter method to obtain the final model. A *P*-value of ≤ 0.05 was statistically significant.

## Results

**Figure 1:**
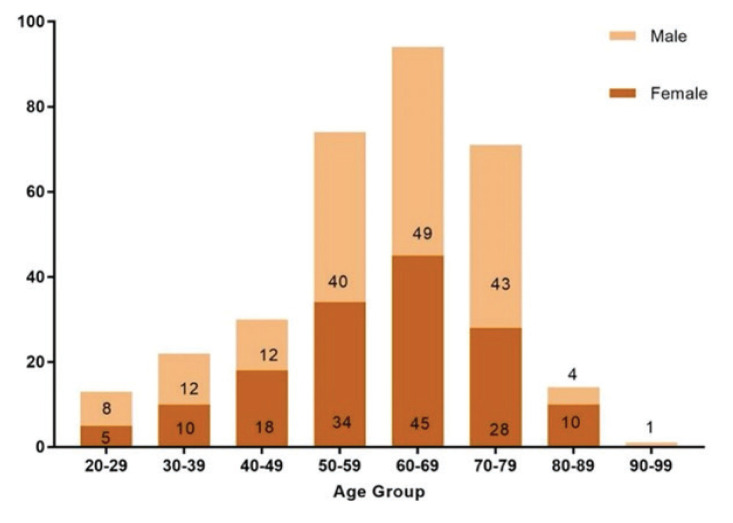
CRC occurrence among YOCRC and AOCRC patients yearly

A total of 319 CRC cases were diagnosed in HUSM between January 2013 and December 2021. AOCRC had 254 patients (79.6%) with a mean age of 65.45 ± 9.10 years old, while 65 patients (20.4%) were YOCRC with a mean age of 38.62 ± 7.67 years old. Most adult patients with CRC were diagnosed in HUSM, with cases being highly represented in the last 4 years, from 2019 to 2021 (Figure 1). Even though YOCRC cases were one-quarter of AOCRC cases, the number of patients identified at a young age in HUSM had gradually increased from 2020 to 2021 (Figure 1).

**Figure 2:**
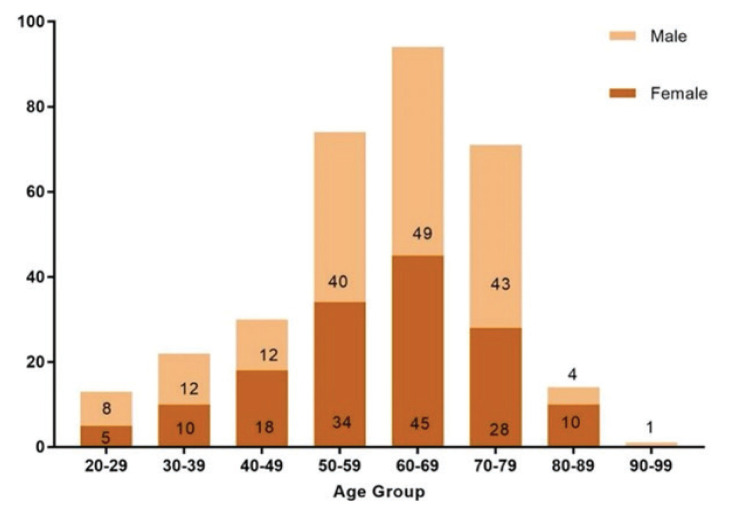
Number of 319 CRC cases with gender per age group among YOCRC and AOCRC

Male patients with AOCRC (53.9%) were higher than female patients with AOCRC (46.1%) (Table 1). Meanwhile, there were no gender differences in YOCRC incidence, with male and female patients with YOCRC accounting for 50.8% and 49.2%, respectively (Table 1). When CRC cases were stratified by gender and age group, female patients with CRC were found to be more prevalent in the younger age range of 40 years old–49 years old, whereas male patients with CRC were more common in the older age groups of 50 years old–59 years old, 60 years old–69 years old and 70 years old–79 years old (Figure 2).

The majority of the Malay population was represented in the AOCRC (90.2%) and YOCRC (97%) (Table 1). In our report, 23.1% of cases of young patients diagnosed with CRC presented with a family history, significantly higher than that of the AOCRC subgroup (9%). Abdominal pain (30.7%) and altered bowel habits (34.3%) were the top two early symptoms among patients with AOCRC. Abdominal pain also was considered the most common initial symptom among patients with YOCRC (41.5%). Most patients with AOCRC and YOCRC were nonsmokers, representing 83.9% and 83.1% of cases, respectively. Meanwhile, diabetes and hypertension were more prevalent in patients with AOCRC than with YOCRC, with 24.4% versus 3.1% and 27.2% versus 7.7%, respectively. Furthermore, most patients in AOCRC and YOCRC both exhibited increased levels of CEA with 57.4% versus 56.9%, respectively.

**Figure 3:**
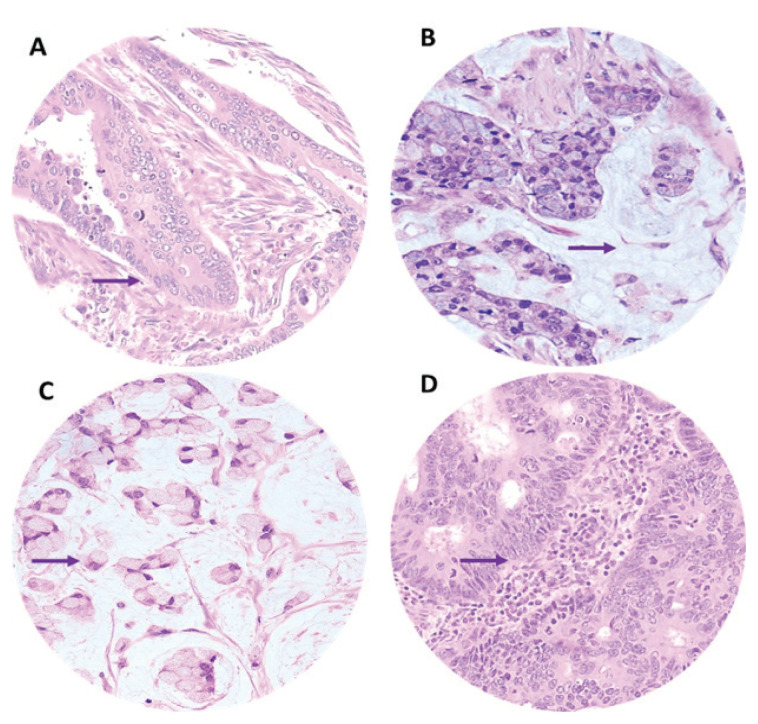
Histological subtypes of CRC with (A) Classical adenocarcinoma - malignant glandular cells arranged in a glandular pattern (arrow) within the desmoplastic stroma. The tumour cells are pleomorphic, having oval to round elongated vesicular nuclei, with some exhibiting prominent large nuclei and abundant eosinophilic cytoplasm. (B) Mucinous adenocarcinoma - malignant tumour cells arranged in clusters and nest patterns with moderate nuclear pleomorphism suspended in pools of extracellular mucin (arrow). (C) Signet ring cells carcinoma - tumour cells arranged in clusters and singly exhibited poorly differentiated signet ring cells with eccentrically placed nuclei (arrow) due to abundant intracytoplasmic mucin. (D) Neuroendocrine carcinoma - malignant glandular cells arranged in a glandular and true resetting pattern

Most AOCRC and YOCRC cases were located on the left side of the colon (85.8% and 83.1%, respectively), primarily at the distal colon (48% and 50.8%, respectively) (Table 2). Adenocarcinomas were found in a larger percentage in AOCRC than YOCRC, with most cases categorised as moderately differentiated subtypes (85.4% versus 64.6%, respectively) (Figure 3A and Table 2).

**Figure 4:**
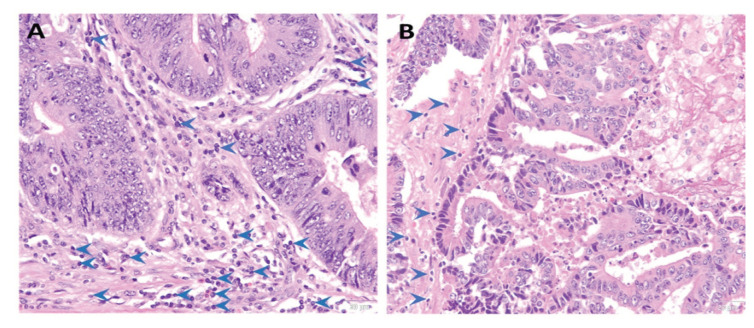
Differences in TILs expression between AOCRC and YOCRC. (A) AOCRC with malignant glands arranged in an irregular glandular pattern exhibiting moderately differentiated adenocarcinoma having high intratumoural lymphocytic infiltrates. (B) YOCRC with moderately differentiated adenocarcinoma comprising malignant glands arranged in a complex glandular, cribriform pattern with low intratumoural lymphocytic infiltrates

Two common colorectal adenocarcinoma variants identified in YOCRC were mucinous adenocarcinoma (15.4%) and signet ring carcinoma (6.1%) (Figures 3B and 3C, Table 2). Another histological subtype observed in AOCRC and YOCRC was neuroendocrine carcinoma (Figure 3D, Table 2). Several patients with AOCRC and YOCRC were diagnosed at advanced stages III and IV (> 65%, Table 2). AOCRC had a high TILs density (61.4%), which was significantly greater than YOCRC (40%) (Figure 4, Table 2).

The multiple LR analysis results (Table 3) showed that three variables (family history of CRC, comorbidity and histological type) were retained in the final model. Those with a family history of CRC were 3.8 times more likely to have YOCRC than those without a family history of CRC (AOR = 3.75, *P* = 0.003). Those with diabetes were 94% less likely to have YOCRC than those without comorbidity (AOR = 0.06, *P* < 0.001) and those with hypertension were 86% less likely to have YOCRC than those with no comorbidity (AOR = 0.14, *P* < 0.001). Patients with a moderately differentiated adenocarcinoma were 15.2 times more likely to have YOCRC than those with a well-differentiated adenocarcinoma (AOR = 15.21, *P* < 0.001). Likewise, patients with a poorly differentiated adenocarcinoma were 4.3 times more likely to have YOCRC than those with a well-differentiated adenocarcinoma (AOR = 4.32, *P* = 0.161). Patients with a mucinous adenocarcinoma were 2.1 times more likely to have YOCRC than those with a well-differentiated adenocarcinoma (AOR = 2.14, *P* = 0.397). Additionally, patients with signet ring carcinoma were 4.5 times more likely to have YOCRC than those with a well-differentiated adenocarcinoma (AOR = 4.52, *P* = 0.100). Finally, patients with neuroendocrine carcinoma were 8% more likely to have YOCRC than those with a well-differentiated adenocarcinoma (AOR = 1.08, *P* = 0.892).

## Discussion

The yearly increase trend of CRC was reported worldwide, including Malaysia (33, 34). In earlier years, CRC had been one of the most prevalent cancers detected in HUSM, a tertiary hospital in Northeastern Malaysia (26). This study highlighted the increase in CRC cases at HUSM, which was more prevalent among older patients than younger patients. Our descriptive data concurred with the increasing trend of AOCRC and YOCRC in three states of Northern Malaysia: Perlis, Kedah and Pulau Pinang (34). Furthermore, male patients with CRC were more common in the older age groups. In contrast, female patients with CRC were more common in the younger age groups, indicating that age and gender are two common variables influencing CRC diagnosis among the local population (35).

Unsurprisingly, Malays accounted for most patients with CRC in HUSM, as this particular ethnicity subgroup also accounted for a larger proportion of the Kelantan population (36). Kelantan was also identified as one of the states in Malaysia with a high prevalence of diabetes, with 47.8% of patients with CRC in HUSM suffering from this metabolic disorder (37, 38). Furthermore, it was shown that patients with diabetes and hypertension in Kelantan were more likely to present with late-stage CRC (37). Our study also added information regarding the prior finding by revealing a significant relationship between a higher prevalence of diabetes and hypertension in patients with AOCRC than those with YOCRC. It was generally established that young patients with a family history of CRC were at a greater risk of developing CRC (39). Our descriptive data revealed that there were more patients with YOCRC with a family history of cancer than patients with AOCRC.

Left-sided CRC is a frequent tumour site among Malaysians and this occurrence links to a pattern in which individuals are presented to the hospital once their cancer has advanced (35, 40). Our findings followed a similar pattern, with moderately differentiated adenocarcinoma being the most prevalent histological form in young and elderly patients. According to our study, the mucinous and signet ring histology types were more aggressive and often associated with a poorer prognosis (35) and more common in the YOCRC group than in the AOCRC group. Most patients with YOCRC had significantly low levels of TILs, indicating immune activation by a tumour. In comparison, most AOCRC showed significantly higher levels of TILs than YOCRC. As TILs density was previously associated with improving disease-specific and overall survival outcomes in patients with CRC (41), more study is required to assess if the trend described in this article could improve the survival outcomes in young and elderly patients with CRC in our local populations.

The multiple LR from our study showed the family history of CRC was a significant factor that increased the likelihood of CRC development in patients with YOCRC with a family history compared to those without one. Additionally, a family history of CRC has a more prevalent impact on YOCRC when compared with AOCRC. The finding agreed with previous studies that also associated family history of CRC as a non-modifiable risk factor for developing CRC in YOCRC (39, 42). According to a report, 30% of YOCRC cases are attributable to a family history of CRC and other inherited diseases (43). We also observed that diabetes and hypertension comorbidities are less likely to cause YOCRC than AOCRC, as these comorbidities are most frequently seen in adult patients with CRC (44). Another significant factor in the CRC development was the histological type from our multiple LR. Moderately differentiated adenocarcinoma is the most general histology (≥ 80%) displayed by patients with CRC; this histology variant accounted for most cases in YOCRC and AOCRC (45). In addition, patients with YOCRC seem to have a stronger predisposition to present with mucinous, signet ring carcinoma and poorly differentiated tumours than AOCRC (46).

We would like to address a few limitations in this study. The study was restricted to a small sample size, which may not represent the total population of Kelantan (28). In addition, the limited sample size in this study reduced the statistical power, possibly influencing the significance of the findings (47). The study only included one tertiary health institution, making it susceptible to a referral bias (48). Furthermore, selection bias may also arise from this retrospective analysis since the criteria used to identify and enroll patients fundamentally differ from those used in previous research cohorts (49, 50). To better understand the CRC burden and compare the characteristics of YOCRC and AOCRC in the Kelantan population, a larger CRC research involving all Kelantan hospitals and correcting those biases would be required.

## Conclusion

Our descriptive study presented the epidemiological and histopathological characteristics of patients with AOCRC and YOCRC diagnosed in HUSM, providing current information on distinguishing features between the groups. Our descriptive data hoped to add some new insights into the clinicopathological aspects of young patients with CRC in the future, therefore supporting patients’ early prognosis.

## Figures and Tables

**Table 1:** Comparing the demographic characteristics between the YOCRC and AOCRC in HUSM

	AOCRC *n* (%)	YOCRC *n* (%)	*P*-value
Gender			
Male	137 (53.9)	32 (49.2)	0.578^a^
Female	117 (46. 1)	33 (50.8)	
Ethnicity			
Malay	229 (90.2)	63 (97.0)	0.57^b^
Chinese	23 (9.0)	1 (1.5)	
Others	2 (0.8)	1 (1.5)	
Family history of CRC			
Absent	231 (91.0)	50 (76.9)	**0.003** a *
Present	23 (9.0)	15 (23.1)	
Initial symptom			
Abdominal distension	19 (7.5)	4 (6.2)	0.452^b^
Abdominal pain	78 (30.7)	27 (41.5)	
Altered bowel habit	87 (34.3)	18 (27.7)	
Per rectal bleeding	70 (27.5)	16 (24.6)	
Smoking status			
Non-smoker	213 (83.9)	54 (83.1)	> 0.99^a^
Smoker	41 (16.1)	11 (16.9)	
Comorbidity			
Absent	123 (48.4)	58 (89.2)	**< 0.001** b *
Diabetes	62 (24.4)	2 (3.1)	
Hypertension	69 (27.2)	5 (7.7%)	
CEA level			
Elevated (≥ 5.2 ng/mL)	146 (57.4)	37 (56.9)	> 0.99^a^
Normal (< 5.2 ng/mL)	108 (42.5)	28 (43.1)	

Notes:

* significant *P*-value;

a analysis by Pearson’s chi-square test;

b analysis by Fisher’s exact test;

CRC = colorectal cancer; YOCRC = young-onset CRC; AOCRC = adult-onset CRC; CEA = carcinoembryonic antigen

**Table 2:** Comparison of clinicopathological characteristics between the YOCRC and AOCRC in HUSM

	AOCRC *n* (%)	YOCRC *n* (%)	*P*-value
Site of the tumour			
Left	218 (85.8)	54 (83.1)	0.695^a^
Right	36 (14.2)	11 (16.9)	
Type of tissue specimen			
Distal colon	122 (48.0)	33 (50.8)	0.680^a^
Proximal colon	36 (14.2)	11 (16.9)	
Rectum	96 (37.8)	21 (32.3)	
Histological type			
Well-differentiated adenocarcinoma	22 (8.7)	5 (7.7)	**< 0.001** b *
Moderately differentiated adenocarcinoma	217 (85.4)	42 (64.6)	
Poorly differentiated adenocarcinoma	6 (2.3)	2 (3.1)	
Mucinous adenocarcinoma	4 (1.6)	10 (15.4)	
Signet ring carcinoma	2 (0.8)	4 (6.1)	
Neuroendocrine carcinoma	3 (1.2)	2 (3.1)	
Staging			
Local (stages I and II)	83 (32.7)	18 (27.7)	0.460^a^
Advanced (stages III and IV)	171 (67.3)	47 (72.3)	
TILs density			
Low	98 (38.6)	39 (60.0)	**0.002** a *
High	156 (61.4)	26 (40.0)	

Notes:

* significant *P*-value;

a analysis by Pearson’s chi-square test;

b analysis by Fisher’s exact test;

YOCRC = young-onset CRC; AOCRC = adult-onset CRC; TILs = tumour infiltrating lymphocytes

**Table 3:** Multiple logistic regression to determine factors contributing more to developing YOCRC than AOCRC

Variables	COR (95% CI)	*P*-value	AOR (95% CI)	*P*-value
Gender				
Male	0.83 (0.48, 1.43)	0.498	-	-
Female	1			
Ethnicity				
Malay	6.33 (0.84, 47.77)	0.074	-	-
Chinese	1			
Others	11.50 (0.51, 261.95)	0.126	-	-
Family history of CRC				
Absent	1		1	
Present	3.01 (1.47, 6.18)	**0.003** *	3.75 (1.55, 9.07)	**0.003** *
Initial symptom				
Abdominal distension	1		-	-
Abdominal pain	1.64 (0.51, 5.26)	0.402	-	-
Altered bowel habit	0.98 (0.30, 3.24)	0.977	-	-
Per rectal bleeding	1.09 (0.33, 3.63)	0.894	-	-
Smoking status				
Non-smoker	1			
Smoker	1.06 (0.51, 2.20)	0.879	-	-
Comorbidity				
Absent	1		1	
Diabetes	0.07 (0.02, 0.29)	**< 0.001** *	0.06 (0.01, 0.26)	**< 0.001** *
Hypertension	0.15 (0.06, 0.40)	**< 0.001** *	0.14 (0.05, 0.40)	**< 0.001** *
CEA level				
Elevated (≥ 5.2 ng/mL)	1			
Normal (< 5.2 ng/mL)	1.02 (0.59, 1.77)	0.935	-	-
Site of the tumour				
Left	1			
Right	1.23 (0.59, 2.58)	0.577	-	-
Type of tissue specimen				
Distal colon	1			
Proximal colon	1.13 (0.52, 2.46)	0.759	-	-
Rectum	0.81 (0.44, 1.49)	0.494	-	-
Histological type				
Well-differentiated adenocarcinoma	1		1	
Moderately differentiated adenocarcinoma	12.92 (3.87, 43.13)	**< 0.001** *	15.21 (3.59, 64.38)	**< 0.001** *
Poorly differentiated adenocarcinoma	3.44 (0.56, 21.25)	0.183	4.32 (0.56, 33.27)	0.161
Mucinous adenocarcinoma	1.72 (0.34, 8.83)	0.514	2.14 (0.37, 12.49)	0.397
Signet ring carcinoma	10.33 (1.83, 58.24)	0.008	4.52 (0.75, 27.26)	0.100
Neuroendocrine carcinoma	1.17 (0.42, 3.28)	0.759	1.08 (0.36, 3.26)	0.892
Staging				
Local (stages I and II)	1			
Advanced (stages III and IV)	1.31 (0.72, 2.40)	0.375		

Notes:

* significant *P*-value;

COR = crude odd ratio; AOR = adjusted odd ratio; CI = confidence interval; CRC = colorectal cancer; CEA = carcinoembryonic antigen
